# Effect of Nano-SiOx/Chitosan Complex Coating on the Physicochemical Characteristics and Preservation Performance of Green Tomato

**DOI:** 10.3390/molecules24244552

**Published:** 2019-12-12

**Authors:** Yingjie Zhu, Dong Li, Tarun Belwal, Li Li, Hangjun Chen, Tingqiao Xu, Zisheng Luo

**Affiliations:** 1 College of Biosystems Engineering and Food Science, Zhejiang Key Laboratory of Agro-Food Processing, Key Laboratory of Agro-Products Postharvest Handling of Ministry of Agriculture and Rural Affairs, Zhejiang University, Hangzhou 310058, China; zhuyingjie@zju.edu.cn (Y.Z.); dong_li@zju.edu.cn (D.L.); tarungbpihed@gmail.com (T.B.); lili1984@zju.edu.cn (L.L.); xutingqiao163@163.com (T.X.); 2 Institute of Food Science, Zhejiang Academy of Agricultural Science, Hangzhou 310058, China; spshangjun@sina.com

**Keywords:** nano-SiOx/chitosan complex coating, green tomato, postharvest quality, microbial load, enzyme activities

## Abstract

A novel nano-silicon oxides (SiOx)/chitosan complex film was prepared using ultrasonic assistant in the process of dissolving chitosan and silicon oxides (SiOx), and characterized by transmission electron microscopy. Its effect on quality preservation of tomatoes (*Solanum lycopersicum* L. cv. Zheza 205) was investigated under ambient temperature. The results revealed that the nano-SiOx/chitosan complex (NSCC) film retarded weight loss and softness, delayed the titratable acids and total soluble solids loss, and thus markedly extended shelf life of green tomatoes. The antimicrobial activity of tomatoes coated with NSCC film was also recorded higher compared to chitosan (Ch) films and control. In addition, the NSCC film-coated tomatoes prevent the increase of malondialdehyde content and total polyphenol content. Moreover, the peroxidase activity, phenylalanine ammonia-lyase activity, and polyphenoloxidase activity of tomatoes coated with NSCC film were found lower than that in other treatments. These data indicated that the beneficial effects of nano-SiOx/chitosan complex coating on postharvest quality were possibly associated with the lower rate of O_2_/CO_2_ transmission coefficient, limiting food-borne pathogenic bacterial growth, higher antioxidant activities, and also higher reactive oxygen species (ROS) scavenging and anti-browning activities of related enzymes in the tomatoes. Further, the results of the study could be used to successfully develop a novel nano-SiOx/chitosan complex film for improving the postharvested quality of tomatoes and thus effectively utilized by the food packaging industry.

## 1. Introduction

Tomato is a kind of climacteric fruit that ripens and deteriorates quickly in uncontrolled storage conditions. To prevent its early deterioration and prolong storage life, green ripen tomatoes are generally harvested and stored to reduce postharvest losses [1]. Postharvest losses reflected in many quality indicators, including the loss of weight and total soluble solids, and the reduction in aromatic compounds. In addition, compared to other fruits and vegetables, post-harvest tomatoes are highly susceptible to foodborne pathogenic bacteria, especially *Escherichia coli* and *Staphylococcus aureus* [2], and therefore pose some serious food safety concerns in public.

Otherwise, a bacterial infection is recognized as one of the external environmental stress which can cause the accumulation of reactive oxygen species (ROS) inside the fruit and the oxidative stress reaction of phenolic compounds [3]. The aging of fruits and vegetables is closely related to the balance of ROS metabolism, which governs the dynamic balance of the production and scavenging of ROS and the synthesis and oxidation of phenolic substances. Excessive ROS can lead to membrane lipid peroxidation, which can cause serious damage to the cell membrane [4]. Phenolic compounds are excellent antioxidant agents, which accumulate in fruits and vegetables to improve antioxidant properties, and their oxidation leads to the common browning phenomenon in fruits and vegetables [5].

For fruits and vegetables, it is advisable to use edible films or coatings to improve the selective permeability of the gas or water, otherwise, the respiratory rate will be enhanced to accelerate the aging process and anaerobic fermentation processes will occur, leading to spoilage [1]. Among others, some biological macromolecules, including polysaccharides, e.g., starches, celluloses, and chitosan, have good coat-forming properties [6,7,8,9]. Among them, chitosan is a natural antimicrobial agent with the advantages of nontoxicity, biodegradability, and wide-spectrum utilization, which is suitable for fruit preservation [10], however, chitosan still has poor mechanical strength [11]. Thus, to improve film properties of chitosan, nanocomposite coatings could be a promising approach. Coatings containing nanoemulsions of citral, nano-titanium dioxide (TiO_2_), nanocomposite Ag/TiO_2_ have recently been proved to be conducive to prolong postharvest shelf-life of fruits like melons and strawberries [12,13,14]. Such beneficial effects could be partly explained by improved physical properties and higher antimicrobial and antioxidant activities [15]. Silicon oxides (SiOx) is one of the excellent nanoparticles that assist in forming a coating film. Due to its polyhydroxy structure, SiOx easily forms hydrogen bonds with the chitosan surface, thereby increasing mechanical properties of the chitosan coating [15]. Recently, SiOx chitosan coatings were applied in the preservation of fresh longan fruits and *Sciaenops ocellatus*, and found superior to single chitosan coating [16,17]. Concerning tomatoes as highly used fruit on a daily basis, very few such nano-coating films have been developed. For instance, Adeshina Fadeyibi and coworkers developed cassava starch-zinc nanocomposite film for providing barrier and mechanical attributes, and thus improved fresh tomatoes quality during storage [18]. Moreover, Patinya Kaewklin and coworkers claimed that chitosan-containing nano-sized titanium dioxide exhibited ethylene photodegradation activity under UV light and consequently delayed the ripening progress of green cherry tomatoes [19]. Still, others found chitosan combined with nano zinc/cerium ion may improve physiological, biochemical, and nutritional parameters of postharvested cherry tomatoes [20]. However, a detailed study on the effect of nano-coating films on tomatoes during storage and especially its effect on fruit quality, enzyme activity, and microbial load is lacking.

With the aim to protect post-harvested tomatoes and increase its storage life, the present study was designed to develop biopolymer films using chitosan with or without nano SiOx and tested for its effectiveness. The overall work consists of three main studies, which includes development of nanofilms and determining its physical properties, resistance to foodborne pathogens, and preservation process.

## 2. Results

### 2.1. Physical Properties and Microstructure

**Figure 1:**
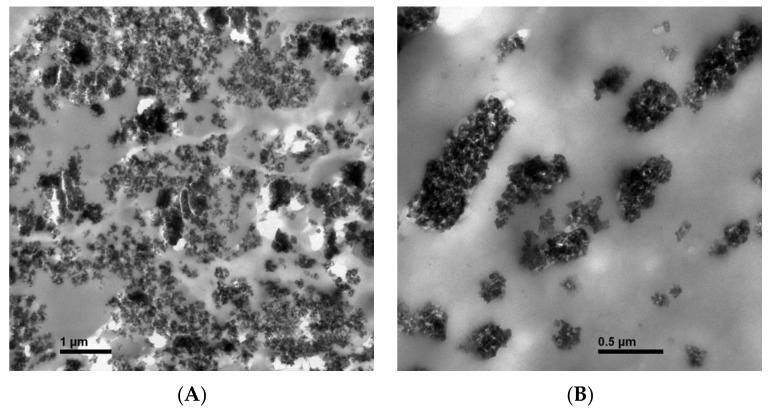
Microstructure of NSCC films with dimensions 1 μm (**A**) and 0.5 μm (**B**) under TEM. NSCC is nano-SiOx/chitosan complex.

As shown in Table 1, the tensile strength of nano-SiOx/chitosan complex (NSCC) film was 124.87 MPa, which was 46.6% higher than the normal chtosan (Ch) film. The light transmittance of NSCC film was also significantly (*p* < 0.05) lower than that of chitosan film. Water vapor transmission rate showed a slight increase from 0.83 Kg/m^2^d to 0.85 Kg/m^2^d after nano-SiOx particles were added into the film. Compared with Ch film, O_2_ transmission coefficient of NSCC film was decreased by 50.0%, while transmission coefficient of CO_2_ was increased by 0.4%, which caused a 33.63% reduction in the rate of Qo_2_/Qco_2_. Magnifying 20,000 times by a transmission electron microscope (TEM), the nano-SiOx particles were seemed to be distributed uniformly in the chitosan film (Figure 1A,B).

Each value represents the mean ± SD of three replicates; different letters in the same column indicate significant difference at *p* < 0.05 according to the least significant difference (LSD) test. Different letters in the same column represents significant difference (*p* < 0.05) according to the least significant difference (LSD) test.

### 2.2. Antimicrobial Activity

Acetic acid solution containing 5.0% (by weight of chitosan) nano-SiOx (NSCC solution) seemed to be much more active among the three solutions. The inhibition zone diameter of 11.52 mm against *Escherichia coli* in case of NSCC was found significantly (*p* < 0.05) higher than Ch and 0.1% acetic acid solution (Control) alone (Table 2, Figure S1). Furthermore, NSCC solution performed better against *Staphylococcus aureus*. The inhibition rate of NSCC solution against *E. coli* and *S. aureus* was 42.88% and 37.85%, respectively, which were significantly (*p* < 0.05) higher than that of Ch solution.

Each value represents the mean ± SD of three replicates; different letters in the same line (for inhibition zone and inhibition rate) indicate significant difference at *p* < 0.05 according to Duncan test. Ch, chitosan. NSCC, nano-SiOx/chitosan complex. Different letters in the same line represents significant difference (*p* < 0.05) according to the least significant difference (LSD) test.

### 2.3. Weight Loss Rate, Firmness, Titratable Acid, Total Soluble Solid, Malondialdehyde, and Total Phenolics Content

**Figure 2:**
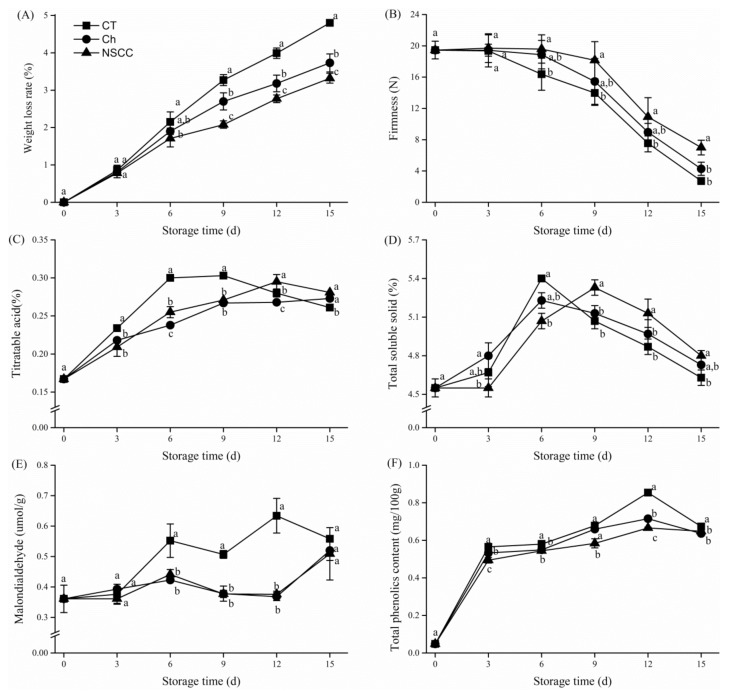
Effects of coating treatments on tomato weight loss (**A**), firmness (**B**), titratable acid (**C**), total soluble solid (**D**), malodialdehyde (**E**), and total phenolics content (**F**) during room temperature storage. CT means control group, Ch means chitosan coating treatment, NSCC means nano-SiOx/chitosan complex coating treatment. Data are mean value ± SD. Different letters under the same storage time significant difference at *p* < 0.05 according to the least significant difference (LSD) test.

It was observed that NSCC coating resulted in lowest weight loss as compared to Ch and control (Figure 2A). The incorporation of nano-SiOx into the chitosan significantly (*p* < 0.05) delayed the weight loss of the fruit, more effectively after 6 days of storage. The firmness of the fruits in each group decreased significantly during the storage period (Figure 2B). However, with NSCC coating, the firmness of tomatoes was recorded highest, which was 157.4% and 63.6% higher than that of control and Ch coating, respectively.

Titratable acidity and total soluble solid are two important flavor indexes. The contents of titratable acids in control fruit increased markedly by 30.3% in the first 9 days and then decreased to 26.1% at the end of storage (Figure 2C). Whereas the contents in Ch- and NSCC-coated tomatoes increased gradually and reached a maximum of 27.3% and 28.1% at the end of storage. Titratable acidity in NSCC-coated tomatoes was significantly higher (*p* < 0.05) than that of Ch-coated tomatoes at day 12, which was found similar to Ch at the last stage of storage. Similar to the changes in titratable acids, a peak point of total soluble solids (TSS) occurred at the 6th day of storage with the value of 5.40% and 5.23% in control and Ch coated tomatoes, respectively, however, it delayed three days in NSCC-coated tomatoes and reached a highest value of 5.33% (Figure 2D). The TSS content in NSCC-coated tomatoes was significantly (*p* < 0.05) higher than that of the control group; however, no significant difference was recorded between NSCC- and Ch-coated tomatoes at the end of storage time.

The degree of lipid peroxidation was estimated as malondialdehyde content (Figure 2E). With the protection of Ch coatings or NSCC coatings, the malondialdehyde content of tomatoes was significantly (*p* < 0.05) lower than the control group at the 6, 9, and 12 days of storage intervals. However, there was no significant (*p* > 0.05) difference between these two groups during the storage. In all conditions, total phenolics content in tomatoes during storage increased dramatically in the first 3 days, followed by a slight increase in the next 9 days and then dropped on the 15th day of storage (Figure 2F). In NSCC-coated tomatoes, total phenolics content showed a more significant (*p* < 0.05) lower value compared with Ch-coated tomatoes.

### 2.4. Antioxidant Enzymes

**Figure 3:**
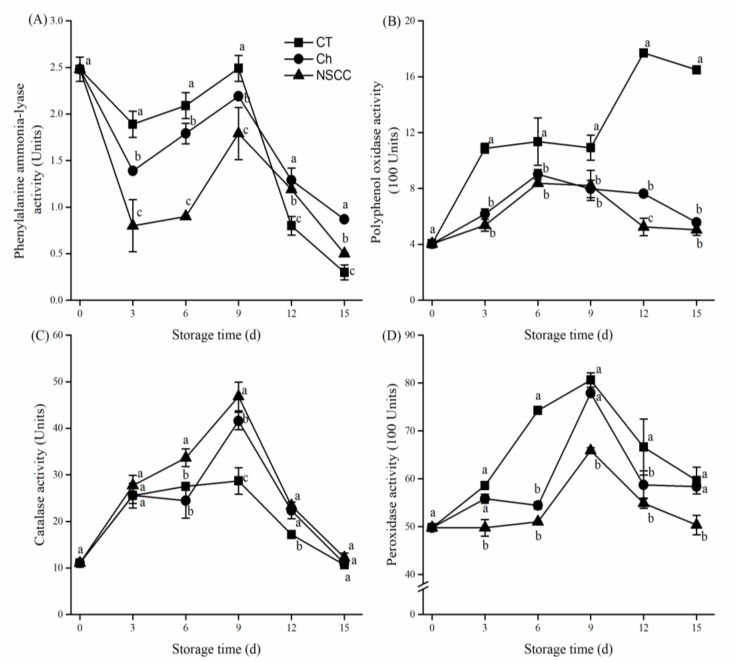
Effects of coating treatments on enzyme activities of (**A**) phenylalanine ammonia-lyase (PAL), (**B**) polyphenol oxidase (PPO), (**C**) catalase (CAT), and (**D**) peroxidase (POD) in tomato fruit during room temperature storage. CT means control group, Ch means chitosan coating treatment, NSCC means nano-SiOx/chitosan complex coating treatment. Data are mean value ± SD. Different letters under the same storage time significant difference at *p* < 0.05 according to LSD test.

Under all treatments, the phenylalanine ammonia-lyase (PAL) activity of tomato decreased first and then increased and decreased during the storage period. After reaching the maximum on the 9th day, the PAL activity decreased sharply (Figure 3A). The PAL activity of Ch- and NSCC-coated tomatoes was significantly different from that of the control group (*p* < 0.05). Precisely, on the day 6th, the PAL activity in the NSCC group significantly decreased by 56.94% and 49.72% compared with the control group and the Ch treatment group, respectively. During the sharp decline period (after the 9th day), the two treatment groups (NSCC and Ch) can alleviate this trend. Polyphenol oxidase (PPO) activity increased slightly in response to Ch coatings and NSCC coatings of tomatoes in the first 6 days after treatment and then decreased almost at the same rate to the level little beyond the initial value in the later stages (Figure 3B). Both coating treatments showed significantly (*p* < 0.05) lower PPO activity as compared to control. However, the NSCC treatment had lower PPO activity compared with Ch treatment during storage time with no significant difference (*p* > 0.05).

Catalase (CAT) activity reached a peak on day 9 and then decreased until the end of storage in all groups. As can be clearly seen that there is no significant difference in CAT activity in all groups at the end of the storage period, whereas NSCC treatment showed significantly higher CAT activity compared to that in the other two groups. Peroxidase (POD) activity showed a similar trend, however, NSCC treatment showed significantly lower POD activity compared to the control group (Figure 3D).

## 3. Discussion

**Figure 4:**
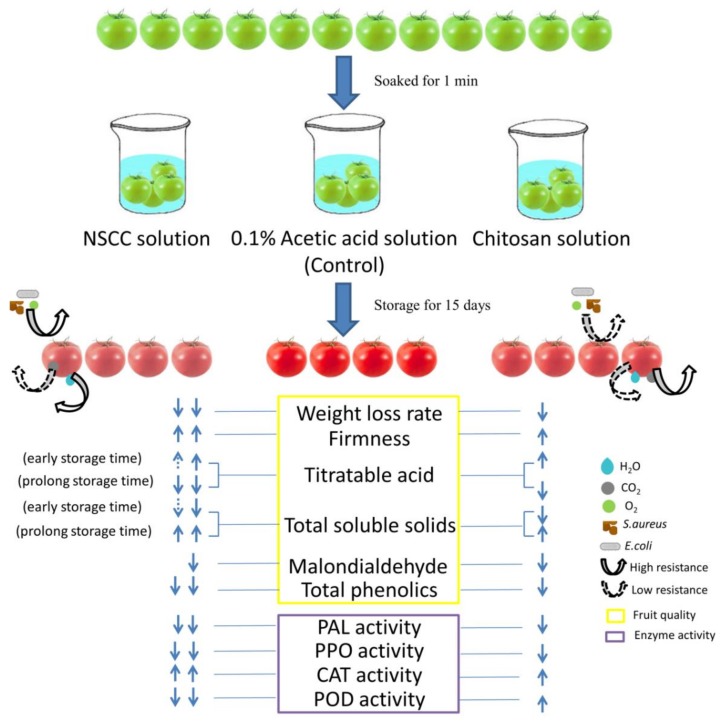
Effect of different coating material on tomatoes fruit quality and enzyme activities.

Nano-chitosan composite coatings applied to postharvest fruit to prolong their shelf life has been concerned in recent years owing to its mechanical and biocompatible properties [21]. In the present study, the NSCC applied over tomatoes was found to improve its overall storage efficiency (Figure 4). Adding nano-SiOx to chitosan could significantly improve its tensile strength, as observed in Table 1. This may be due to the formation of stronger hydrogen bonds with the addition of nano-SiOx in chitosan [17]. The significantly lower light transmittance by the NSCC coating indicates higher surface and interface roughness, and smaller pore size [22], which may also explain the reduction rate of water vapor transmission and Qo_2_/Qco_2_ as compared to Ch film. The lower water vapor transmission correlated with lower weight loss [23], and this can also be seen in the present study. In addition, the higher CO_2_ transmission coefficient and lower O_2_ transmission coefficient during NSCC coating create a better microenvironment with low oxygen and high carbon dioxide to reduce fruit respiration rate and further delay senescence [24].

Limiting microbial load is also a critical factor to delay senescence and increase the shelf life of the fruits [25]. The antibacterial activity of the coating treatments was analyzed against two typical foodborne pathogenic microbes, *E. coli* and *S. aureus*. The inhibition rate of NSCC solution against *E. coli* and *S. aureus* was 42.88% and 37.85%, respectively, which were significantly (*p* < 0.05) higher than that of Ch solution (Table 2). This may be due to the fact that free chitosan contains amino acid group on its surface [26], thus limits its antibacterial activity. It has also been suggested that the antibacterial activity depends on the ionization balance of chitosan [27]. In the present study, when SiOx dissolves in chitosan solution, a weakly acidic environment was formed, which may prompt protonation of its amine function group, and thus resulted in higher antibacterial activity. Similar improvements in antibacterial activity were reported elsewhere. For instance, Zhang et al. [28] reported that adding nano TiO_2_ into pure chitosan resulted in the highest antimicrobial activity against *E. coli* with the bactericidal rate over 99.9%. Overall, nano-SiOx/chitosan films overcome inadequacies of pure chitosan and enhanced the antimicrobial activity.

Postharvest maturity and senescence cause flavor loss and mostly increased water evaporation rate, softening and decreased ratio of sugar to acid content. Fruit weight loss is affected by many external conditions outside preservation film on fruit surface and film properties themselves as well. The former include temperature and relative humidity, etc., and the latter may be attributed to the pore size and homogeneity of the film or hydrogen bond formation rate on the interface of preservation film and fruit surface or within the film, etc. [1]. NSCC-coated tomatoes showed the lowest weight loss among all the treatments and also delayed the process of weight loss which may be better relate to the lower water vapor transmission rate under NSCC film. The firmness of the tomato fruits decreased rapidly during post-maturation period in the control group, while significantly higher firmness in NSCC-coated tomatoes was recorded, which was consistent with the results reported by Zhang et al. [29]. The titratable acid increase during early fruit maturity mainly attributed to the accumulation of acids and subsequent decrease can be related to the higher respiration and ripening rate where organic acids could be used as a substrate in respiration process or to their conversion to sugars [30]. In the present study, the semipermeable NSCC film prevents respiration during the storage period. This was reflected by lower respiration rate in the early maturity time to delay organic acids synthesis and also lower respiration rate in the later maturity stages to delay organic acids bioconversion in the NSCC compared to Ch film and control group [30]. There was a synergistic relationship between the changes in TSS and titratable acids, and in the early storage time, soluble carbohydrates were mainly accumulated [31]. In prolong storage time, sugar was consumed to maintain metabolism, hence a decline in TSS was recorded. In the present study, NSCC-coated tomatoes showed comparable higher TSS content and hence slowed down the maturity rate as compared to the control (*p* < 0.05). This indicated that NSCC film can effectively delay the aging process and prevent the decrease in TSS content of tomatoes during storage period, which was similar to the earlier study on using ε-polylysine/chitosan film coating in citrus preservation [32].

Edible coatings enhanced the fruit quality by reducing the moisture loss and gas exchange, as well as by reducing the pathogenic microorganism attack and its deterioration [33]. In the present study, we mainly focused on the ROS stress damage and phenolic antioxidant accumulation during post-maturity period of tomato fruits under different coatings. Synthesis of phenolic compounds in plants increased after injury, pathogen attack, or during the stress conditions [34]. The level of total phenolic compounds could be considered as a marker other than antioxidant enzymes for the defense response in plants against environmental stress. NSCC coating resulted in marked decreases in total phenolic content significantly throughout the whole storage period. Many other studies reported that physical treatment like hyperbaric or low doses of UV irradiation promoted the accumulation of phenolic compounds in tomatoes [35,36,37]; however, in the present study, NSCC coating treatment did not show any additional abiotic stresses on tomatoes. It was also reported that the production of ROS induced lipid peroxidation that resulted in membrane injury and increase the MDA level [38,39]. MDA is an index to test lipid peroxidation and could be considered to represent the level of oxidative stress. The significantly lower level of MDA in NSCC- and Ch-coated tomatoes than the control group during high respiration stage (6th–12th days of storage) indicated that the two edible films can protect the fruit from environmental stress as well [40].

Further reasons for the improvement of oxidative stress ability are probably related to the enzyme activities. Phenylalanine ammonia-lyase (PAL) is a key enzyme in the phenylpropanoid pathway responsible for phenolic compounds synthesis [41,42]. Polyphenol oxidase (PPO) can catalyze the hydroxylation reaction of free phenolic acids in fruits and vegetables, along with the dehydrogenation reaction of hydroxyphenols to hydrazine and the reaction of hydrazine with intracellular proteins or self-condensation in fruits and vegetables to produce brown pigment or melanin [43]. Thus, the activities of the two enzymes mainly affected the content of phenolic compounds in fruits. The PAL activity of the tomato fruits treated with NSCC coating film increased slowly and it was always lower than the control group and the Ch treatment group (Figure 3A), which is consistent with the change in the total phenolic content (Figure 2F). The lower PPO activity in Ch and NSCC treatment (Figure 3B) indicated that both coating films have certain effects on inhibiting the browning of tomatoes, of which the NSCC coating film has a stronger effect. PPO activity is affected by O_2_ concentration and phenolic concentration [44]. Since the better O_2_ transmission coefficient and slower phenolic accumulation was recorded in NSCC coating film, the lower PPO activity seems logical. Catalase (CAT) and peroxidase (POD) are antioxidant enzymes that prevent the formation of reactive oxygen species (ROS) [45]. In the present study, these antioxidant enzymes level increased under environmental stress and gradually decreased when the stress reached a certain level (Figure 3C,D), hence stimulate the fruit aging process, losing its sensory properties and nutrients [46]. The NSCC treatments induced higher CAT activity in the green tomatoes than the Ch treatment and control, indicating the role of NSCC coating in delaying senescence of tomatoes. However, lower activity of POD in NSCC-coated tomatoes was recorded in the present study. The possible reason could be due to the lower response against oxidative stress since oxidative stress products like MDA and antioxidant compounds like phenolics all showed a lower level in NSCC coated tomatoes. Li et al. also observed lower activity of POD when investigated the effect of nano-powder polyethylene packing on fresh strawberries harvested during green stage [47].

## 4. Materials and Methods

### 4.1. Preparation of Chitosan (Ch) and Nano-SiOx/Chitosan Complex (NSCC) Films

In a preliminary orthogonal experiments, the ultrasonic time, as well as the amount of chitosan (food grade, deacetylation degree ≥ 95%, Zhejiang golden-shell biochemistry Co. Ltd., Taizhou, China) and SiOx (average particle size is 20–50 nm, Zhejiang Hong Sheng material technology Co. Ltd., Hangzhou, China) added into the complex film were optimized. It was found that the combination of 20 g chitosan and 1 g nano-SiOx during a 10 min of ultrasonication (DS-8510DHT; 40 KHz, 600W Shanghai Sonxi ultrasonic instrument Co., Ltd., Shanghai, China) in a 2 L system produced the best NSCC film in term of higher tensile strength.

Thus, to obtain Ch solution, 20 g chitosan was pre-dissolved in 200 mL of 1% acetic acid (*v*/*v*) (Aladdin Industrial Co., Shanghai, China) and diluted with distilled water to a final volume of 2 L. The mixture was then stirred for 10 min at 120 rpm using electronic blender (OES20; 60W Wenzhou Biaonuo instrument Co., Zhejiang, China) while keeping it in ultrasonic bath. To obtain NSCC solution, the same system with added 1 g of SiOx was ultrasonicated for 10 min along with stirring at 120 rpm to evenly dispersed SiOx.

Samples of Ch and NSCC films were prepared by freezing the prepared solutions with liquid nitrogen and sliced into pieces of 100 nm thickness (Leica EM UC, Wetzlar, Germany).

### 4.2. Evaluation of the Microstructures and Physical Properties of Ch and NSCC Films

#### 4.2.1. Water Vapor Transmission Rate (P)

Water vapor transmission rate was determined in a 25 × 40 mm weighing bottle in the presence of 3 g anhydrous CaCl_2_. The sealed bottle was placed in a dryer containing saturated NaCl solution. The relative humidity and the temperature in the dryer were maintained at 75% and 25 °C, respectively. The weight of the weighing bottle was measured at intervals of 24 h until the weight change tends to be stable. Water vapor transmission rate (*P*) was calculated as follows [48] (Equation (1)):

(1) $$P=\left(W_{f}-W_{i}\right)/\left(d*S\right)$$

where *W_f_* is the final weight of the weighing bottle, *W_i_* is the initial weight of the weighing bottle, *S* is the active area of the coating film, and *d* is test days.

#### 4.2.2. O_2_ Transmission Coefficient

A 50 mL tripod bottle containing 10 mL oleic acid was sealed with the film and placed in a dryer at room temperature. The system was first balanced under N_2_ environment for one day and then replaced with O_2_. The partial pressure of O_2_ outside of the film was maintained at 1 atm. The O_2_ transmission coefficient of the film was calculated as follows [49] (Equation (2)):

(2) $$Q_{o2}=\Delta m/\Delta t$$

where *Q_O_*_2_ is the O_2_ transmission coefficient (g/d); Δ*t* is the time taken to increase weight (d); Δ*m* is the weight of O_2_ absorbed by oleic acid (g).

#### 4.2.3. CO_2_ Transmission Coefficient

A 50 mL conical flask containing 20 g of KOH sealed with NSCC film was put in a dryer at room temperature. The system was first balanced under N_2_ environment for one day and then permuted with CO_2_. The partial pressure of CO_2_ outside of the film was maintained at 1 atm. The CO_2_ transmission coefficient of the film was calculated as follows [49] (Equation (3)):

(3) $$Q_{co2}=\Delta m/\Delta t$$

where *Qc_O_*_2_ is the CO_2_ transmission coefficient (g/d); Δ*t* is the time taken to increase weight (d); Δ*m* for the weight of CO_2_ absorbed by KOH (g).

#### 4.2.4. Measurement of Light Transmittance

The adsorption value of NSCC film was detected by ultraviolet spectrophotometer (UV-1750, Shimadzu, Japan) set at a wavelength of 450 nm. The light transmittance (*T*) was calculated as shown in Equation (4) [50]:

(4) $$T\;\left(\%\right)\;=10^{-A}$$

where *A* represents the adsorption value.

#### 4.2.5. Tensile Strength

The tensile strength was determined using an auto tensile tester (XLW, Labthink Ltd., Jinan, China) with a 500 N load cell. The films were cut into rectangle strips (120 mm × 12 mm). The initial distance between the grips was 90 mm, and the strain rate was 300 mm/min. The tensile strength of tested films was determined by calculating the mean of three replicates.

#### 4.2.6. Transmission Electron Microscopy

The microstructure of the surfaces of NSCC films was examined using a transmission electron microscope (JEM-1200EX, JEOL Ltd., Tokyo, Japan). Dispersion quality of nanomaterials into the complex film matrix was monitored. The distribution uniformity was verified by comparison with a rectangular coordinate system containing 240 randomly drawn points.

### 4.3. Antimicrobial Activity

#### 4.3.1. Bacterial Strains

The strains included in this research (*Escherichia coli*, CICC23657 and *Staphyloccocus aureus*, CICC10384) were provided by the microbiology laboratory of the Department of Food Science and Nutrition, Zhejiang University, Hangzhou, China.

#### 4.3.2. Preparation of Bacterial Culture

*Staphylococcus aureus* (*S. aureus*) and *Escherichia coli* (*E. coli*) strain were firstly cultured on Agar medium. Standard plating medium was prepared by adding 10 g of special peptone (Sigma-Aldrich Canada Co., Oakville, ON, Canada), 15 g of granulated agar (Sigma-Aldrich Canada Co., Oakville, ON, Canada), 5 g of sodium chloride (Sinopharm Chemical Reagent Co., Ltd., Shanghai, China), 5 g of yeast extract (Sigma-Aldrich Canada Co., Oakville, ON, Canada), and 5 g of beef extract (Sinopharm Chemical Reagent Co., Ltd., Shanghai, China) in one liter of water and the pH was adjusted to 7.2 with NaOH. The bacteria cultures were incubated at a constant temperature incubator of 37 °C for 18 h and then placed in physiological saline of 10 mL to prepare bacteria suspension before being used for spread plate assays.

#### 4.3.3. Determination of Inhibition Zone

Agar diffusion assay (Inhibition test) was conducted in aseptic conditions using sterile 1/4-in. (0.635-cm)-diameter paper disks (WH10328170, Schleicher & Schuell, Inc., Keene, NH, USA). Briefly, 1 mg acetic acid was added into 100 mL blended liquid transferred from 2 L Ch or NSCC solution (as both explained in 4.1), respectively, to form the testing solutions (treatments). For the agar diffusion assay, 0.1% (*v*/*v*) acetic acid tested groups were kept as control.

0.2 mL diluted bacteria suspension for 10^6^–10^7^ cfu/mL uniformly swabbed on the agar medium. 3 pieces of sterile disks were placed on the medium with tweezers after 10 min, and then the three tested solutions (20 μL for each) were loaded on the placed sterile discs in order. After being incubated at 37 °C for 22 h, the growth of bacterial colonies on petri plates was observed by measuring the diameter of the inhibition zone. Each inhibition zone was equally divided into six pieces, which were measured sequentially with a Vernier caliper and the mean value was taken. All experiments were repeated in triplicates. The results were expressed as the mean diameter of inhibition zone in mm ± standard deviation (mean ± SD). Ch treatment and NSCC treatment were evaluated for antimicrobial efficacy by calculating the indicator of inhibition rate (*IR*) (Equation (5)) [51].

(5) $$\begin{array}{ll} IR\left(\%\right) & =(Inhibition\;zone\;diameter_{treatment}\\ & -Inhibition\;zone\;diameter_{control})\\ & /\;Inhibition\;zone\;diameter_{treatment}\;\times \;100 \end{array}$$

### 4.4. Plant Material and Sampling

Tomato fruits (*Solanum lycopersicum* L. cv. Zheza 205) at green ripe stage were purchased from vegetable base in Wuxing District, Huzhou, China. The fruits were sorted to eliminate physical damage or pathological defects and selected for uniform size and color, and then randomly divided into three groups each containing 150 fruits. Tomato fruits were respectively impregnated in Ch solution (mentioned at Section 4.1), NSCC solution (mentioned at Section 4.1), and distilled water containing 0.1% (*v*/*v*) acetic acid (control group, CT) for 1 min. They were all put in plastic crates after drying in cooling air and stored at an artificial climate chamber (PRX-450B, Saifu, Ningbo, China) to maintain a constant RH of 86% at 23 ± 1 °C. Samples were taken initially and at each 3-day intervals during 15 days of total storage time to test related indicators. Each treatment has three replicates.

### 4.5. Weight Loss Rate, Firmness, Titratable Acid, Total Soluble Solid, Malondialdehyde, and Total Phenolics Content

Fifteen tomatoes were randomly selected, and divided into 3 groups (Ch, NSCC, CT) containing 5 fruits each. Fruits were weighed using a precision balance (Mettler AE200, Giessen, Germany) during the storage period and at each 3-day intervals for 15 days. The weight loss was indicated by the rate of decrease in the weight of the tomato on each storage day compared to the weight measured on the first day.

Firmness was measured at three different positions of the equatorial region on each fruit (eight tomatoes for each replicate) using a texture analyzer (TA-XT2i, Stable Micro Systems Ltd., Godalming, UK) with a 5 mm diameter probe. Fruits were penetrated up to 5 mm at a rate of 0.5 mm/s and the maximum force was recorded and expressed in Newton (N).

Titratable acid content (TA) was determined by titrating 10 mL tomato juice with 0.1 mol/L sodium hydroxide solution and expressed as citric acid (mass/mass) on the basis of tomato weight. To calibrate the original CO_2_ contained in the system, phenolphthalein indicator was added and neutralized with 0.1 mol/L sodium hydroxide solution until the color turns red.

Total soluble solids value in tomato juice samples was determined using a refractometer (Minolta) in triplicate as described by Luo et al. [52].

The malondialdehyde (MDA) content in tomato samples was determined using the thiobarbituric acid method [53]. Briefly, ground tomato samples (5 g) were homogenized in 10 mL Phosphate Buffered Saline (PBS) buffer. After centrifugation at 12,000× *g* for 20 min, 4 mL aliquot of the supernatant was mixed with an equal volume of 20% TCA containing 0.5% thiobarbituric acid (TBA, volume setting with 10% TCA). The mixture was boiled in a water bath for 30 min. The absorbance was recorded at 532 and 600 nm. The absorbance at 600 nm was read to correct for unspecific turbidity. The amount of MDA formed was calculated using extinction coefficient of 155 mM^−1^ cm^−1^ and expressed as μmol·g^−1^ FW. Three biological replicates were carried out for each experiment.

Total phenolics content in tomato juice samples was determined by adopting Folin–Ciocalteu assay method [54]. Briefly, 5 g of the fruits were ground with 10 mL 80% ethanol and then centrifugated at 3500 r/min for 20 min. An aliquot of 0.5 mL of the supernatant was added to 0.5 mL of Folin–Ciocalteu reagent and left for 3 min. Thereafter, 10 mL of saturated sodium carbonate solution was then added and the volume brought up to 25 mL with distilled water. Results were calculated on fresh weight basis (f.w.) as mg Gallic acid equivalents (GAE) per gram of the sample.

### 4.6. Phenylalanine Ammonia-Lyase (PAL), Polyphenol Oxidase (PPO), Catalase (CAT), and Peroxidase (POD) Activity

For PAL assay, about 5 g of frozen samples were homogenized in 10 mL 0.05 mol/L PBS buffer (pH 6.8) containing 6 g polyvinylpyrrolidone, 5 mmol/L β-mercaptoethanol, and 2 mol/L EDTA according to the method of Cheng and Breen [55]. The homogenate was centrifuged for 15 min at 14,000× *g* and the supernatant collected for the determination of enzyme activity. PAL (EC 4.3.1.5) activity was determined as described by Luo et al. [56] with minor modifications. Briefly, an aliquot (500 μL) of the extract was incubated with 0.5 mL of 0.02 mol/L-phenylalanine and 3 mL of 0.05 mol/L boric acid buffer (pH 8.8) at 30 °C for 1 h, and then 0.1 mL 6 mol/L hydrochloric acid was used to stop the reaction, after which absorbance at 290 nm was measured. One unit of PAL activity was expressed as a change of 0.01 in OD_290_ g^−1^ FW min^−1^.

For PPO assay, tissue (5.0 g) from six slices were homogenized in 10 mL of 0.05 mol/L phosphate buffer (pH 6.8). The homogenate was centrifuged at 10,000× *g* for 20 min at 4 °C and the supernatants were collected as enzyme extracts. PPO (EC1.10.3.1) activity was assayed according to the method of Jiang [57] by measuring the oxidation of 2.8 mL 0.1% (*w*/*v*) 4-methylcatechol using 0.2 mL enzyme extract. The increase in absorbance at 420 nm was automatically recorded for 3 min, using a spectrophotometer (UV-1750, Shimadzu, Japan). One unit of PPO activity was defined as a change of 0.01 in OD_420_ g^−1^ FW min^−1^.

CAT activity was measured according to Luo et al. [58] protocol. Briefly, frozen samples (5.0 g) were ground in 10 mL of 0.05 mmol/L sodium phosphate buffer (pH 7.8) at 4 °C. After centrifuging at 10,000× *g* at 4 °C for 20 min, the supernatant was collected as the crude enzyme extract for assay. The assay mixture (3 mL) was comprised of 400 μL enzyme extract, 600 μL H_2_O_2_ (10 mM), and 3 mL 50 mM phosphate buffer. One unit of POD activity was defined as a change of 0.01 in OD_240_ g^−1^ FW min^−1^.

POD (EC 1.11.1.7) activity was determined by the method described by Luo et al. [59] with slight modification. Briefly, frozen samples (5.0 g) were ground in 20 mL of 0.05 mol/L sodium phosphate buffer (pH 6.8) containing 1% (w/v) polyvinylpyrrolidone at 4 °C. After centrifuging at 12,000× *g* at 4 °C for 10 min, the supernatant was collected as the crude enzyme extract for assay. The reaction mixture contained 2.4 mL 0.05 mol/L potassium phosphate buffer (pH 6.5), 0.25 mL 0.16 mol/L guaiacol and 0.1 mL enzyme extract in 3 mL assay volume, and the reaction was initiated by adding 0.25 mL of 0.88 mol/L H_2_O_2_. The increase in absorbance at 470 nm was recorded. One unit of POD activity was defined as a change of 0.01 in OD_470_ g^−1^ FW min^−1^.

### 4.7. Statistical Analysis

The experiments were laid out in a completely randomized design. SPSS 20.0 software (SPSS Inc., Chicago, IL, USA) was used for data analysis. Data were expressed as mean ± standard deviation (SD) and were analyzed using analysis of variance (ANOVA), and the means were compared at significance level (*p* < 0.05) by the least significant difference (LSD). Graphs were plotted using the OriginPro 9.1 software (OriginLab, Northampton, MA, USA).

## 5. Conclusions

In the present study, the nano-SiOx/chitosan complex coating film was successfully developed and applied on tomatoes for testing its preventive effect under storage condition. The formation of semipermeable barriers by nano-SiOx/chitosan complex coating successfully maintains the quality of tomatoes by slowing down moisture loss, gas exchange, and respiration rates. Further limiting bacterial growth, flavor loss, and physiological disorders development, especially reflected in retaining phenolic compounds and improving ROS oxidative stress ability inside tomato bodies, were also recorded. Moreover, nano-SiOx/chitosan complex coating does not induce high enzymatic activities of all investigated antioxidant enzymes, hence delay tomato senescence.

Overall the results of the present study clearly showed the positive influence of introducing nano-SiOx to form nano-SiOx/chitosan complex coating, which successfully protected the tomatoes and improved its shelf-life. From both the commercial and economical point of view, these results could be of significance for the food packaging industries. Further in-depth examinations should be conducted regarding the influence of composite coating on the related enzyme activities of tomatoes by understanding their gene expression, aiming to develop coating agents with better, prolonged effects on tomatoes.

## Figures and Tables

**Table 1:** Physical properties of chitosan (Ch) film and nano-SiOx/chitosan complex (NSCC) film.

Physical Properties	Water Vapor Transmission Rate (kg/m^2^ d)	O_2_ Transmission Coefficient (g/d)	CO_2_ Transmission Coefficient (g/d)	Light Transmittance (%)	Tensile Strength (MPa)
Ch film	0.85 ± 0.01 ^a^	0.0015 ± 0.0002 ^a^	3.0769 ± 0.0015 ^b^	66.03 ± 2.93 ^a^	85.17 ± 1.39 ^b^
NSCC film	0.83 ± 0.00 ^a^	0.0010 ± 0.0001 ^b^	3.0905 ± 0.0021 ^a^	30.65 ± 4.60 ^b^	124.87 ± 6.28 ^a^

**Table 2:** The effect of nano-SiOx/Chitosan complex solution on inhibition zone diameter and inhibition rate of different bacteria.

Bacterial Strains	Inhibition Zone Diameter (mm)			Inhibition Rate (%)	
	Acetic Acid Solution (Control)	Ch Solution	NSCC Solution	Ch Solution	NSCC Solution
*Escherichia coli*	6.58 ± 0.02 ^c^	9.40 ± 0.04 ^b^	11.52 ± 0.09 ^a^	30.00 ± 0.09 ^b^	42.88 ± 0.27 ^a^
*Staphylococcus aureus*	7.80 ± 0.01 ^c^	9.68 ± 0.03 ^b^	12.55 ± 0.12 ^a^	19.45 ± 0.21 ^b^	37.85 ± 0.51 ^a^

## Supplementary Materials

The following are available online. Figure S1: The effect of nano-SiOx/Chitosan complex solution on inhibition zone diameter of different bacteria.
