# Supplementary material for: Effect of Nano-SiOx/Chitosan Complex Coating on the Physicochemical Characteristics and Preservation Performance of Green Tomato
Source: Molecules. 2019 Dec 12;24(24):4552. doi: 10.3390/molecules24244552 (PMC6943560; doi:10.3390/molecules24244552)
Supplement: Supplementary file 1 [file molecules-24-04552-s001.pdf]

# Figure S1.

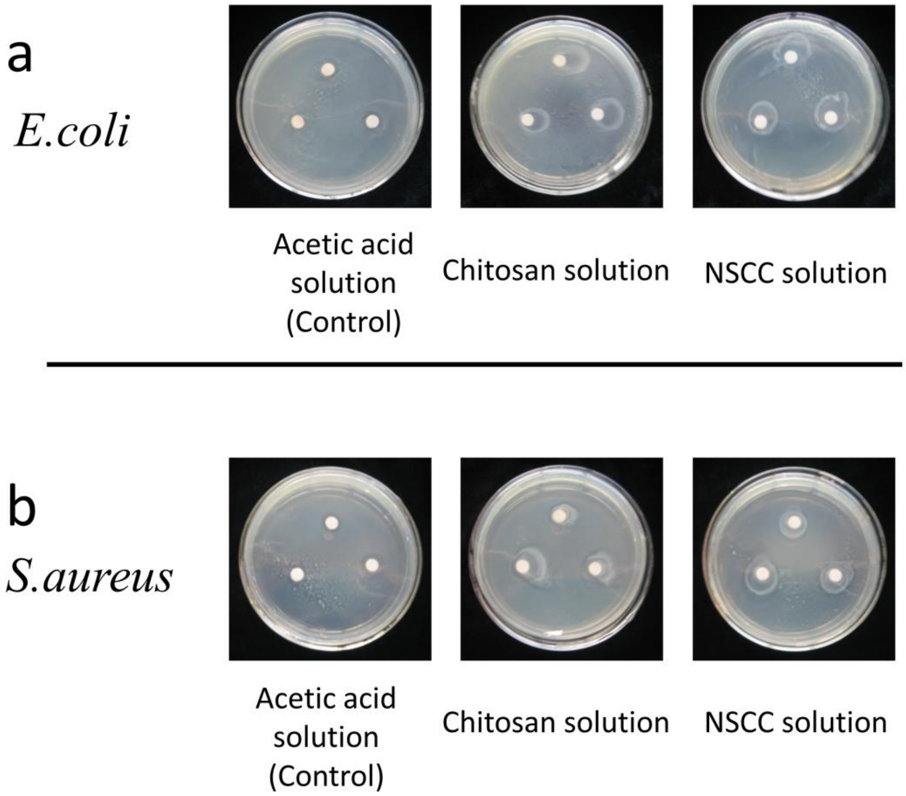


**Figure S1.** The effect of nano-SiOx/Chitosan complex solution on inhibition zone diameter of different bacteria.
